# Assessing Hemorrhagic Shock Severity Using the Second Heart Sound Determined from Phonocardiogram: A Novel Approach

**DOI:** 10.3390/mi13071027

**Published:** 2022-06-28

**Authors:** Yan Chen, Aisheng Hou, Xiaodong Wu, Ting Cong, Zhikang Zhou, Youyou Jiao, Yungen Luo, Yuheng Wang, Weidong Mi, Jiangbei Cao

**Affiliations:** 1 Department of Anesthesiology, the First Medical Center of Chinese PLA General Hospital, Beijing 100853, China; yanzicw@126.com (Y.C.); houaisheng301@163.com (A.H.); wuxiaodong@301hospital.com.cn (X.W.); drcontigo@163.com (T.C.); zhouzhikang0906@163.com (Z.Z.); jyy18131375045@163.com (Y.J.); yungenluo@126.com (Y.L.); wwdd1962@163.com (W.M.); 2 The Faculty of Electrical Engineering and Computer Science, Ningbo University, Ningbo 315211, China; nano.plasma.uv@gmail.com

**Keywords:** hemorrhagic shock, hypotension, the second heart sound, phonocardiogram, electrocardiogram

## Abstract

Introduction: Hemorrhagic shock (HS) is a severe medical emergency. Early diagnosis of HS is important for clinical treatment. In this paper, we report a flexible material-based heart sound monitoring device which can evaluate the degree of HS through a phonocardiogram (PCG) change. Methods: Progressive hemorrhage treatments (H1, H2, and H3 stage) were used in swine to build animal models. The PCG sensor was mounted on the chest of the swine. Routine monitoring was used at the same time. Results: This study showed that arterial blood pressure decreased significantly from the H1 phase, while second heart sound amplitude (S2A) and energy (S2E) decreased significantly from the H2 phase. Both S2A and S2E correlated well with BP (*p* < 0.001). The heart rate, pulse pressure variation and serum hemoglobin level significantly changed in the H3 stage (*p* < 0.05). Discussion: The change of second heart sound (S2) was at the H2 stage and was earlier than routine monitoring methods. Therefore, PCG change may be a new indicator for the early detection of HS severity.

## 1. Introduction

Hemorrhagic shock (HS) is one of the leading causes of death after trauma, which is caused by the depletion of intravascular volume through blood loss to the point of being unable to match the tissue’s demand for oxygen [1,2]. There are multiple systems and indicators for a clinical diagnosis of HS. In the physical diagnosis, the most helpful indicators are either severe postural dizziness or a large postural pulse change [3]. In massive transfusion scores evaluation, both hypotension and tachycardia are used. Further, the shock index (SI, heart rate [HR]/systolic blood pressure [SBP]) ≥ 1 can also be used to identify patients with critical bleeding and serve as a trigger for the initiation of a prehospital blood transfusion [4,5]. However, the traditional diagnostic indicators have a certain delay; the changes of indicators only appear when the amount of bleeding reaches a certain level. This fact delays the medical decisions of the doctor and the rescue of HS patients.

In recent years, the multi-physiological signal method has been used to determine the degree of blood loss. For example, the combination of Doppler and electrocardiogram (ECG) signals has been used to classify the severity of bleeding [6,7]. Compared to the traditional monitoring methods, the multi-physiological signal method has the characteristics of fast, non-invasive, convenient, continuous monitoring. These characteristics are necessary for a HS diagnosis and clinical rescue. Among the multi-physiological signals, a phonocardiogram (PCG) is an accurate indicator of cardiac system examination [8,9]. PCG signal is the indicator of acoustic information from the contraction and relaxation of the heart, and it can be used to assess left ventricular function and detect changes in contractility [10,11]. 

Heart sounds are the noises that are generated by the beating heart and the resultant flow of blood through it. The second heart sound (S2) is a component of the heart sound. S2 has two audible components, the aortic closure sound and the pulmonic closure sound. Therefore, S2 is produced in part by hemodynamic events immediately following the closure of the aortic and pulmonic valves. The vibrations of the second heart sound occur at the end of ventricular contraction and identify the onset of ventricular diastole and the end of mechanical systole. In recent years, many studies about PCG have shown that the second heart sound spectral-frequency features can be used to estimate the mean arterial pressure (MAP) and pulmonary artery pressure [12,13]. As a result, the heart sound signal-based method for continuous blood pressure (BP) measurement is a promising method for assessing blood loss [14,15].

During the progress of hemorrhagic shock, effective circulatory blood-volume reduction leads to significant arterial pressure decreases. Although other studies have proved that the second heart sound has a good correlation with the BP [14,16], the correlation between the S2 and HS stages is still unknown. Therefore, this study aimed to prove that the S2 can be a diagnostic indicator for the severity of HS. At the same time, we report a wearable device that can contain synchronized analysis PCG. With the help of this device, we also aimed to evaluate if PCG can be a more efficient indicator for HS diagnosis, which may provide a new method for clinical diagnosis.

## 2. Materials and Methods

### 2.1. Animals

This study was performed in eight healthy male Bama swine (weighted 23.98 ± 1.63 kg, aged 8 to 10 months). All operations follow the Guide for the Care and Use of Laboratory Animals published by the National Institutes of Health. The Medical Ethics Committee of the Chinese People’s Liberation Army General Hospital approved the protocol for this experimental study (approval number: 2020-X16-27).

### 2.2. Anesthesia and Surgical Procedures

Swine were sedated with an intramuscular injection of midazolam (0.3 mg/kg) and xylazine hydrochloride (0.03 g/kg) and then intubated. Anesthesia was maintained with sevoflurane (1–1.5%). Intravenous rocuronium (1 mg/kg) was administrated for mechanical ventilation (Ohmeda Excel210SE, Ohmeda Drive, Madison, WI, USA). The serum hemoglobin (Hb), glucose (Glu), and lactate (Lac) levels were detected using an ABG analyzer (GEM Premier 3000, Instrumentation Laboratory Company, Hartwell Avenue Lexington, Lexington, MA, USA). All swine were laid in the supine position. After local infiltration anesthesia of lidocaine (2%), two catheters were inserted into the right carotid artery and jugular vein via the right cervical incision to allow monitoring of the blood pressure and central venous pressure (CVP), respectively. Another catheter was advanced into the right femoral artery via puncture guided by portable ultrasound for blood extraction. Nasopharynx temperature and pulse oxygen saturation (SpO2) were measured by a multifunctional monitor (Mindray Bene VisionN17, Mindray Medical International Limited, Shenzhen, China).

### 2.3. Experimental Design

**Figure 1:**
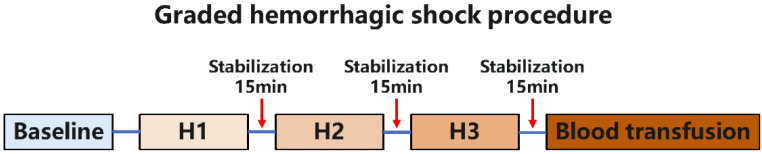
Graded hemorrhagic shock and resuscitation protocol. BL: baseline; H1: the first stage of hemorrhage with 5 mL/kg of blood accumulated; H2: the second stage of hemorrhage with 10 mL/kg of blood accumulated; H3: the third stage of hemorrhage with 20 mL/kg of blood accumulated; T: the last stage of transfusion of autologous blood.

The cardiopulmonary signals (ECG, heart rate, carotid pressure, SpO2, tidal volume, respiratory frequency, airway pressure) and nasopharyngeal temperature of the swine were monitored continuously during the experiment, and the baseline values were recorded simultaneously. Then, a three-stage graded hemorrhage and resuscitation protocol was conducted (H1, H2, and H3 stage) [17]. The release of 5 mL/kg of blood was performed in the first hemorrhage step (H1), followed by 15 min of stabilization. Subsequently, the release of another 5 mL/kg of blood (cumulative 10 mL/kg) was performed in the second hemorrhage step (H2). After 15 min of stabilization, the release of 10 mL/kg of blood (cumulative 20 mL/kg) was performed in the third hemorrhage step (H3), again followed by 15 min of stabilization (Figure 1). The total blood loss reached nearly 30% of the estimated whole blood volume of a swine, which could result in severe HS. All extracted blood was preserved in a blood-stored bag with an anticoagulant agent. No fluids or vasoactive agents were administrated for volume compensation or blood pressure elevation during the hemorrhage stage. After the graded hemorrhage protocol, transfusion of autologous blood was executed, and a blood transfusion was completed in 30 min. The animals were euthanized with an intravenous injection of potassium chloride after the experiment.

### 2.4. Heart Sound Signal Collection

**Figure 2:**
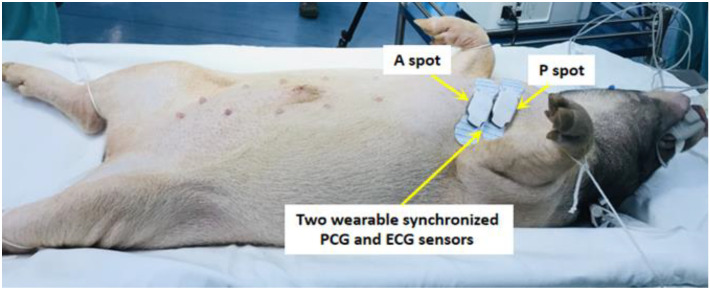
Two wearable synchronized phonocardiogram (PCG) and electrocardiogram (ECG) sensors are attached to the aortic valve second auscultation area (A spot) and pulmonary auscultation area (P spot) on the swine’s left chest surface, respectively.

Two wearable synchronized PCG and ECG sensors developed by Wenxin Tech (Fuzhou, China) were attached to the aortic valve second auscultation area (A spot) and the pulmonary auscultation area (P spot) on the left chest surface of the swine, respectively (Figure 2). The device includes a reusable centerpiece and two one-time disposable patches. The patches are the electrodes for the single-lead ECG, and a sound sensor is located at the center of the reusable piece. Both the heart sound and ECG signals can be detected by the device synchronously, and all data that are acquired from the device can be transferred to a computer via Bluetooth and then transferred to a cloud-based data center for storage and analysis.

### 2.5. PCG Data Analysis

**Figure 3:**
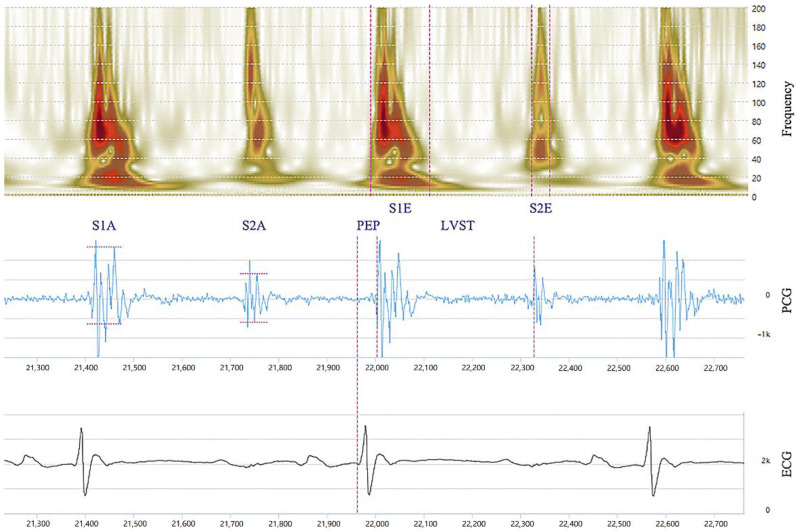
The parameters measured are marked on a combination of ECG, PCG, and frequency: the first sound amplitude (S1A); the first sound energy (S1E); PEP (the time period from the start of the ECG Q peak to the S1E peak); the second sound amplitude (S2A); the second sound energy (S2E); LVST (the time period from the start of the S1E peak to the S2E peak).

After data cleansing, the heart sounds were extracted and separated into the S1 and S2. The characteristics of S1 and S2 signals, including the first sound amplitude (S1A); the first sound energy (S1E); the pre-ejection period (PEP); the second sound amplitude (S2A); the second sound power (S2E); and the left ventricular systolic time (LVST) were measured using an automatic analysis software that was developed by Bayland Scientific and Wenxin Tech (Beijing, China). The parameters (S1A, S2A, S1E, S2E, PEP, LVST) that were marked on a combination of ECG, PCG, and frequency are shown in Figure 3. In this study, the device can record frequency, PCG and ECG synchronously. The frequency part is the frequency response of the PCG signal. The frequency response is obtained by wavelet transform. The sound spectrogram (frequency axis) is a visual representation of an acoustic signal, with degrees of amplitude at various frequencies by time. A 10 s, recording of the heart sounds was taken as a discrete data point for analysis, and a time-frequency analysis using wavelet transform offers better insight into this compounded effect. Measurements for each index of the heart sound were the average values that were measured in all heart cycle periods every 10 s.

### 2.6. Statistical Analysis

All continuous variables are expressed as mean ± standard deviation (mean ± SD). ANOVA was used to compare the hemodynamic and heart sound parameters variation in different hemorrhagic shock and resuscitation steps. The statistical differences between any two steps were analyzed by an LSD (least significant difference) post hoc test. A bivariate linear correlation (Pearson) was used to test the relationship between the heart sound parameters and circulatory variables. SPSS software, version 22.0 (SPSS, Inc., Chicago, IL, USA) was used to perform the statistical analysis.

## 3. Results

### 3.1. PCG Sensor Working Process

**Figure 4:**
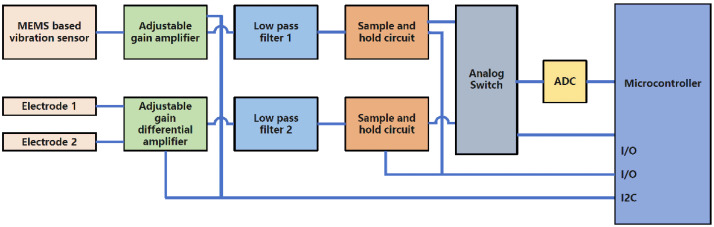
The block diagram of synchronized ECG and PCG. The MEMS-based vibration sensor is used to detect PCG signals. The adjustable gain amplifier is used to adjust the gain and it is controlled by microcontroller firmware. The PCG signal passes a low pass filter to eliminate high-frequency noises. On the ECG side, two electrodes are used to collect ECG signals. The ECG signals pass through the adjustable gain differential amplifier. The gain is controlled by microcontroller firmware. The ECG signal passes through a low pass filter to eliminate high-frequency noise. To achieve the simultaneous collection of both ECG and PCG, two samples and hold circuits are employed. The two sample and hold circuits are controlled by one digital output from the microcontroller. The microcontroller uses one digital output to sample and hold both ECG and PCG analog signals. After sampling and holding the ECG and PCG, an analog switch is employed to select ECG or PCG to pass ADC to digitize the analog signal.

The monitoring of systemic blood volume by PCG requires the coordination of a series of cardiac hemodynamic indicators and cardiac electrical activity. Therefore, the PCG sensor and ECG sensor need to work synchronously. PCG was used as the main indicator to evaluate systemic blood volume, and ECG was used as the synchronous processing indicator. The signal acquisition and processing of the PCG sensor and ECG sensor are shown in Figure 4.

### 3.2. Routine Monitoring Indicators during Graded Hemorrhagic Shock

**Figure 5:**
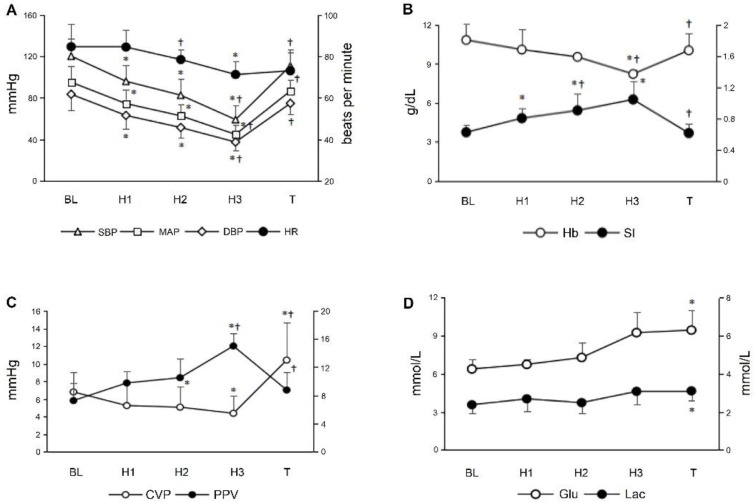
Changes in the hemodynamic and metabolic variables during graded hemorrhagic shock and resuscitation. (**A**) Blood pressure-related variables and heart rate decreased significantly. SBP: systolic blood pressure; MAP: mean arterial pressure; DBP: diastolic blood pressure; HR: heart rate. (**B**) Hemoglobin decreased significantly with the graded hemorrhagic shock. The shock index increased significantly with the graded hemorrhagic shock. Hb: hemoglobin; SI: shock index. (**C**) Central venous pressure decreased significantly with the graded hemorrhagic shock. The pulse pressure variation increased significantly with the graded hemorrhagic shock. CVP: central venous pressure; PPV: pulse pressure variation. (**D**) Glucose and lactate were significantly increased at autologous blood transfusion stage. Glu: glucose; Lac: lactate. * Significant difference from the baseline (*p* < 0.05); ^†^ significant difference from the preceding step of protocol (*p* < 0.05).

Changes in the SBP, MAP, DBP, HR, SI, CVP, pulse pressure variation (PPV), Hb, Glu and Lac during graded hemorrhagic shock and resuscitation are shown in Figure 5. Stepwise hemorrhage results in progressive decreases in SBP, MAP, DBP in the H1 (95.88 ± 15.13 vs. 120.13 ± 16.82, *p* = 0.003; 74.38 ± 13.16 vs. 95.0 ± 15.35, *p* = 0.005; 63.0 ± 13.05 vs. 83.38 ± 15.14, *p* = 0.009, respectively); H2 (82.5 ± 15.88 vs. 120.13 ± 16.82, *p* = 0.007; 62.63 ± 11.01 vs. 95.0 ± 15.35, *p* = 0.011; 51.38 ± 9.88 vs. 83.38 ± 15.14, *p* = 0.012, respectively); and H3 (59.0 ± 13.3 vs. 120.13 ± 16.82, *p* < 0.001; 44.75 ± 9.33 vs. 95.0 ± 15.35, *p* < 0.001; 37.38 ± 7.74 vs. 83.38 ± 15.14, *p* < 0.001, respectively) stage. The SI progressively increased in the H1 (0.86 ± 0.04 vs. 0.71 ± 0.06, *p* = 0.010); H2 (1.01 ± 0.01 vs. 0.71 ± 0.06, *p* = 0.015); and H3 (1.19 ± 0.15 vs. 0.71 ± 0.06, *p* = 0.020) stages. The CVP had significant decreases in the H2 and H3 stages (5.05 ± 2.33 vs. 6.78 ± 2.21, *p* = 0.046 and 4.35 ± 2.03 vs. 6.78 ± 2.21, *p* = 0.003, respectively). However, the HR, PPV and Hb significantly changed in the H3 stage (71.2 ± 6.34 vs. 84.6 ± 10.9, *p* = 0.024; 15.0 ± 1.83 vs. 7.25 ± 2.5, *p* = 0.033 and 8.20 ± 0.30 vs. 10.82 ± 1.20, *p* = 0.060, respectively). The serum Glu and Lac levels had significant elevation in the last transfusion stage (9.43 ± 1.60 vs. 6.37 ± 0.78, *p* = 0.044 and 3.10 ± 0.51 vs. 2.37 ± 0.42, *p* = 0.029, respectively). The transfusion of autologous blood resulted in partial hemodynamic variables, including HR, SBP, MAP, DBP, SI, PPV and Hb restored toward baseline values (all *p* > 0.05).

### 3.3. PCG and Associated Indicators during Graded Hemorrhagic Shock

**Figure 6:**
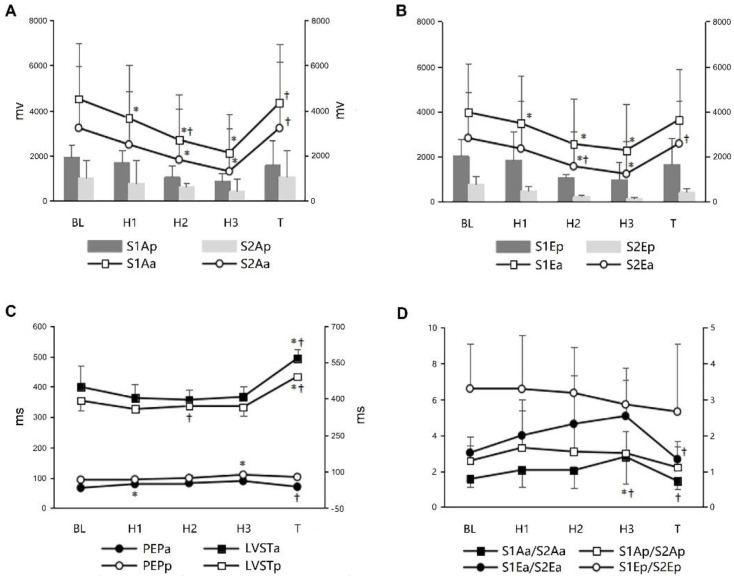
Changes in the S1Aa, S1Ap, S2Aa, S2Ap, S1Ea, S1Ep, S2Ea, S2Ep, PEPa, PEPp, LVSTa, LVSTp, S1Aa/S2Aa, S1Ap/S2Ap, S1Ea/S2Ea and S1Ep/S2Ep during graded hemorrhagic shock and resuscitation. (**A**) The amplitude of S1 and S2 decreased significantly with the graded hemorrhagic shock at A and P spot. S1Aa: S1A at the A spot; S1Ap: S1A at the P spot; S2Aa: S2A at the A spot; S2Ap: S2A at the P spot; (**B**) The energy of S1 and S2 decreased significantly with the graded hemorrhagic shock at A and P spot. S1Ea: S1E at the A spot; S1Ep: S1E at the P spot; S2Ea: S2E at the A spot; S2Ep: S2E at the P spot; (**C**) The pre-ejection period and left ventricular systolic time changes at A and P spot. PEPa: PEP at the A spot; PEPp: PEP at the P spot; LVSTa: LVST at the A spot; LVSTp: LVST at the P spot. (**D**) S1/S2 ratio changes of amplitude and energy at A and P spot. * Significant difference from the baseline (*p* < 0.05); ^†^ significant difference from the preceding step of protocol (*p* < 0.05).

Changes in the S1A (S1Aa); S1E (S1Ea); S2A (S2Aa); S2E (S2Ea); PEP (PEPa); andLVST (LVSTa) at A spot, and S1A (S1Ap); S1E (S1Ep); S2A (S2Ap); S2E (S2Ep); PEP (PEPp); and LVST (LVSTp) at P spot during graded hemorrhagic shock and resuscitation are shown in Figure 6. Significantly progressive decreases in the S1Aa and S1Ea were found in the H1 (3654.38 ± 2360.31 vs. 4504.63 ± 2735.63 mv, *p* = 0.013, and 3475.88 ± 2091.99 vs. 3963.13 ± 2157.06, *p* = 0.012, respectively); H2 (2691.75 ± 2280.63 vs. 4504.63 ± 2735.63 mv, *p* = 0.004 and 2551.63 ± 2020.05 vs. 3963.13 ± 2157.06, *p* = 0.011, respectively); and H3 (2122.63 ± 1917.82 vs. 4504.63 ± 2735.63 mv, *p* = 0.006 and 2276.0 ± 2068.78 vs. 3963.13 ± 2157.06, *p* = 0.018, respectively) stage. The S2Aa and S2Ea significantly decreased in the H2 (1812.5 ± 2006.32 vs. 3224.13 ± 2455.87 mv, *p* = 0.023 and 1562.5 ± 1557.84 vs. 2818.63 ± 2040.51, *p* = 0.015, respectively) and H3 (1298.88 ± 1687.52 vs. 3224.13 ± 2455.87 mv, *p* = 0.020 and 1223.25 ± 1447.81 vs. 2818.63 ± 2040.51, *p* = 0.017, respectively) stage. The S1Aa/S2Aa had significant decreases in the H3 stage (2.80 ± 1.47 vs. 1.56 ± 0.44, *p* = 0.048). However, no significant changes in the S1Ap, S2Ap, S1Ep, S2Ep, S1/S2Ap and S1/S2Ep were observed throughout the protocol (all *p* > 0.05). The PEPa significantly elevated in the H1 stage (79.0 ± 2.0 vs. 66.25 ± 8.02, *p* = 0.039), whereas the PEPp elevated in the H3 stage (87.0 ± 4.0 vs. 66.33 ± 4.62, *p* = 0.004). The autologous blood transfusion caused a restoration in the S1Aa, S2Aa, S1Ea, S2Ea, PEPa and PEPp to baseline values (all *p* > 0.05).

### 3.4. Correlations between PCG Associated Indicators and Routine Monitoring Indicators

**Figure 7:**
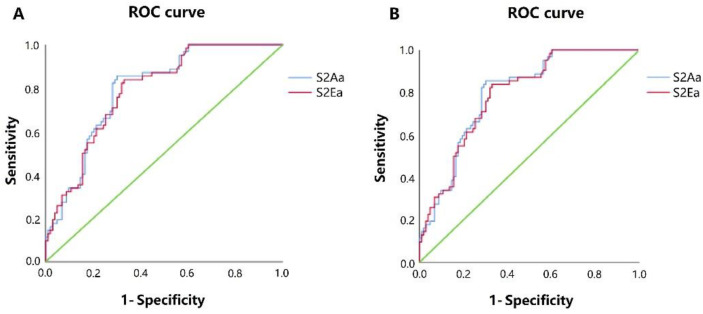
(**A**) ROC curve analysis showed that the sensitivity and specificity of S2Aa ≤ 251.5 mv for the diagnosis of SBP < 90 mmHg were 0.968% and 0.417%, respectively, and the sensitivity and specificity of S2Ea ≤ 243 for the diagnosis of SBP < 90 mmHg were 0.984% and 0.408%, respectively. The AUC was 0.795 for S2Aa and 0.788 for S2Ea. (**B**) ROC curve analysis showed that the sensitivity and specificity of S2Aa ≤ 204.5mv for the diagnosis of MAP < 60 mmHg were 0.930% and 0.354%, respectively, and the sensitivity and specificity of S2Ea ≤ 182 for the diagnosis of MAP < 60 mmHg were 0.942% and 0.354%, respectively. The AUC was 0.705 for S2Aa and 0.707 for S2Ea. ROC: receiver operating characteristic curve; AUC: area under the curve.

Correlations between the heart sound parameters and circulatory variables are shown in Table 1. There were good correlations between ΔS2Aa, ΔS2Ea, ΔS1Aa/S2Aa, ΔS1Ea/S2Ea and ΔSBP (r = 0.519, *p* < 0.001; r = 0.559, *p* < 0.001; r = −0.550, *p* < 0.001; r = −0.526, *p* < 0.001, respectively); ΔMAP (r = 0.511, *p* < 0.001; r = 0.532, *p* < 0.001; r = −0.523, *p* < 0.001; r = −0.509, *p* < 0.001, respectively); and ΔDBP (r = 0.478, *p* < 0.001; r = 0.480, *p* < 0.001; r = −0.503, *p* < 0.001; r = −0.489, *p* < 0.001, respectively). There were strongly negative correlations between the ΔLVSTa and ΔHR, ΔSI, and ΔPPV (r = −0.693, *p* < 0.001; r = −0.620, *p* < 0.001, and r = −0.519, *p* < 0.001, respectively). On the other hand, both ΔS1Aa and ΔS1Ea did weakly positive correlate with the ΔSBP, ΔMAP and ΔDBP, respectively (all *p* < 0.05). A receiver operating characteristic (ROC) curve analysis showed that the sensitivity and specificity of S2Aa ≤ 251.5 mv for the diagnosis of SBP < 90 mmHg were 0.968% and 0.417%, respectively, and the sensitivity and specificity of S2Ea ≤ 243 for the diagnosis of SBP < 90 mmHg were 0.984% and 0.408%, respectively. The area under the curve (AUC) was 0.795 (95% confidence interval 0.728–0.862; *p* < 0.001) for S2Aa and 0.788 (95% confidence interval 0.721–0.856; *p* < 0.001) for S2Ea. The sensitivity and specificity of S2Aa ≤ 204.5 mv for the diagnosis of MAP < 60 mmHg were 0.930 and 0.354, respectively, and the sensitivity and specificity of S2Ea ≤ 182 for the diagnosis of MAP < 60 mmHg were 0.942 and 0.354, respectively. The AUC was 0.705 (95% confidence interval 0.624–0.785; *p* < 0.001) for S2Aa and 0.707 (95% confidence interval 0.627–0.787; *p* < 0.001) for S2Ea (Figure 7).

## 4. Discussion

In this study, we compare the differences and correlations between PCG-associated indicators and routine monitoring indicators in different stages of hemorrhagic shock. The result shows that except for BP and SI, other hemodynamic variables, including HR, CVP, PPV, and Hb change significantly, starting with the H2 or H3 hemorrhage stage. Serum Glu and Lac levels increase in the last transfusion stage. However, the S1A, S1E, S2A, and S2E that are determined from the PCG decrease consistently with the BP drop in this acute HS model, and both the ΔS2A and ΔS2E correlate well with the ΔBP. More importantly, the S2A or S2E contributes to the diagnosis of SBP < 90 mmHg or MAP < 60 mmHg, and changes earlier than HR, PPV, Hb, or metabolic variables. Additionally, the LVST has good correlations with the SI or PPV; therefore, the S2 signal might be valuable in the early detection of BP drop that is induced by blood loss and might be used to assess hemorrhage severity.

In our study, the results show a good correlation between the S2A or S2E and the BP. Despite previous research proposing the determination of BP by heart sound analysis, BP estimation from the heart sound has only achieved progress in recent years [14,18,19]. Early studies suggest that a unique noninvasive BP measurement and a small range of BP variations may induce bias [16]. With the progress of sensory techniques, the characteristics of S1 and S2 signals are connected to SBP and DBP using a neural network algorithm, and heart sound-based BP estimation presents a reliable, non-invasive, and continuous alternative to existing methods of continuous BP measurement. Kapur et al. reported that the heart sound-based method with regularization using a piezo-accelerometer chest sensor with a computer model could provide accurate BP monitoring (SBP ranged from 58 to 173 mmHg) in critically ill children [15]; however, the findings were limited by age, pathophysiologic differences, or other conditions leading to abnormal heart sounds. Peng et al. reported on BP estimation using heart sound signals that were recorded by the microphone of a smartphone [14], but they used a cold pressor stimulus to increase the BP and environmental noise interfered with the measurements. In previous studies, separated acoustic sensors and ECG electrodes were used to detect signals from PCG and ECG, respectively [20]. More recently, several similar products comprising a PCG and ECG monitoring system have been invented, e.g., a smart stethoscope system. The wearable synchronized PCG and ECG sensor used in our study can evaluate the left ventricular systolic function and contribute to the diagnosis of heart failure [10]. Our study demonstrated that decreased S1 and S2 signals were synchronized with the BP drop (SBP ranged from 37 to 142 mmHg) that was induced by acute and progressive blood loss, suggesting that the heart sound may potentially be used to reflect the severity of hemorrhage.

The heart sound signals that are obtained from the aortic valve or pulmonary auscultation spot are changeable, suggesting that the heart sound variation may be site-dependent. This would require an accurate site of patching and allow for a professional or trained person to use the small device. Bodo et al. found that the sequence of vital signs cessation was respiration, brain electrical activity [21], heart sound, BP, and ECG in a swine model of lethal hemorrhage. Our study also found that the S2 had a trend (but not statistically significant) of a greater degree of decline than that of BP, and this might partly explain why the cessation of the heart sound occurred before BP. The S2 was of a smaller amplitude during acute blood loss; however, the situation may be not like that during chronic blood loss. For example, in anemic patients, the aortic component of the S2 was of a greater amplitude, and the accentuated S2 might result from the lower blood viscosity [22]. Additionally, the results of this self-control study regarding relative variation in heart sound signals are applicable for abnormal heart sounds that are caused by valve disease, dextrocardia, and other conditions.

Aside from BP and HR, Hb is another important variable in several massive transfusion scores [23]. This study also showed that the serum Hb concentration significantly decreased in the severe hemorrhage stage. The blood lactate/peripheral perfusion ratio was a marker for predicting mortality in animals undergoing acute hemorrhage, and admission blood glucose predicted the incidence of hemorrhagic shock in multiple trauma, non-diabetic patients [24,25]. However, our study revealed that significant changes were observed in serum Lac or Glu levels in the last transfusion stage instead of the hemorrhage stage, indicating that the metabolite variables cannot be the early indicators of the acute HS. While the findings of our study still need to be confirmed in clinical trials, this study could possibly expand the potential clinical application of a wearable PCG device in monitoring hemorrhage in high-risk patients in many prehospital settings (such as traffic accidents, natural disasters, and intentional or unintentional injury).

The PPV is one of the gold standards for the prediction of fluid responsiveness; however, it significantly increases in the severe hemorrhage step in this study. The PEP is the time period from the start of the ECG Q peak to the opening of the aortic valve within the S1 heart sound, and it depends mainly on preload. Bendjelid et al. proposed that the ΔPEP that was produced by positive pressure ventilation was useful in predicting the fluid-responsiveness of patients in shock [26]. Our study also showed that ΔPEP increased in the H1 stage after bleeding, and the LVST had a good correlation with the PPV. Thus, the PEP or LVST might be a potential indicator of hypovolemia.

In clinical rescue, any hemodynamic variable may play an important role in the treatment of hemorrhagic shock because hemodynamic variables can guide the amount and type of fluid replacement in hemorrhagic shock treatment. In our study, the PCG-based S2 monitoring can provide the key hemodynamic variable and assess hemorrhagic shock earlier. The early diagnosis of hemorrhagic shock is of great significance for clinical rescue. In addition, this wearable PCG monitoring device also has the potential to be more flexible and more miniaturized, as well as the potential for subcutaneous implantation. With micro-scale energy harvesting technology [27,28], this device may achieve continuous power supply and uninterrupted monitoring, which is of great significance for clinical treatment and the early detection of diseases.

As exploratory research, our study also has some limitations. First, we did not measure the cardiac output (CO), stroke volume (SV), stroke volume variation (SVV), and systematic vascular resistance (SVR) because of limited conditions in the animal laboratory. The correlations between the S2A or S2E and CO, SV, SVV, and SVR are unavailable. Further research is needed to assess the accuracy of BP estimation by PCG analysis in clinical use. Second, since decreased BP leads to a decrease in amplitude and frequency of the heart sound, when there is no BP drop due to chronic or small amounts of bleeding or cardiovascular compensation in awake patients, the S1 or S2 change might be insignificant. With more detailed research on heart sound features, whether the S1 or S2 distinguish the BP drop that is caused by blood loss or other reasons needs to be further investigated. Third, it is hard to keep both PCG and ECG signals measured at their standard positions and leads simultaneously due to the curved surface of the swine body. Lastly, other factors such as heart sound detection errors may induce bias.

## 5. Conclusions

Our study provides novel and converging cardio-physiological evidence that hemorrhage-induced BP drop can produce a heart sound decrease synchronously, which occurs earlier than HR, CVP, PPV, Hb, or metabolic variables. The S2A or S2E correlates well with the BP. It has high sensitivity in the diagnosis of SBP < 90 mmHg, implying that the S2 may serve as a potential candidate for cardio-physiological markers of HS. The S2 signal might be capable of promptly determining the degree of blood loss indirectly, which would provide a valuable means for real-time remote triage and decision making. Therefore, the S2 might be a new, earlier marker for predicting hemorrhagic shock than the other parameters that are evaluated in this study. Further, although the simpler, small patch is site-dependent, it is easy to be handled by a trained person. While the findings of our study need to be confirmed in the clinical setting, they do suggest a role for the clinical application of wearable PCG monitoring in high-risk trauma patients in the prehospital setting.

## Figures and Tables

**Table 1:** Correlations between the heart sound parameters and circulatory variables.

	ΔHR	ΔSBP	ΔMAP	ΔDBP	ΔSI	ΔPPV	ΔCVP
ΔS1Aa							
r	0.045	0.241	0.210	0.189	−0.271	−0.349	0.279
*p*	0.574	0.002	0.008	0.018	0.001	<0.001	0.001
ΔS2Aa							
r	−0.036	0.519	0.511	0.478	−0.179	−0.442	0.227
*p*	0.652	<0.001	<0.001	<0.001	0.025	<0.001	0.007
ΔS1Ea							
r	−0.006	0.254	0.225	0.210	−0.369	−0.154	0.105
*p*	0.941	0.001	0.005	0.008	<0.001	0.100	0.220
ΔS2Ea							
*r*	−0.160	0.559	0.532	0.489	−0.363	−0.380	0.161
*p*	0.045	<0.001	<0.001	<0.001	<0.001	<0.001	0.060
ΔS1Aa/S2Aa							
*r*	0.252	−0.550	−0.523	−0.503	0.455	0.117	−0.092
*p*	0.001	<0.001	<0.001	<0.001	<0.001	0.211	0.283
ΔS1Ea/S2Ea							
*r*	0.237	−0.526	−0.509	−0.489	0.448	0.098	−0.200
*p*	0.003	<0.001	<0.001	<0.001	<0.001	0.294	0.019
ΔPEPa							
*r*	−0.397	−0.469	−0.509	−0.509	−0.105	0.170	−0.331
*p*	<0.001	<0.001	<0.001	<0.001	0.192	0.068	<0.001
ΔLVSTa							
*r*	−0.693	0.358	0.276	0.170	−0.620	−0.519	0.320
*p*	<0.001	<0.001	<0.001	0.033	<0.001	<0.001	<0.001

Relative variation in S1Aa (ΔS1Aa); S2Aa (ΔS2Aa); S1Ea (ΔS1Ea); S2Ea (ΔS2Ea); S1Aa/S2Aa (ΔS1Aa/S2Aa); S1Ea/S2Ea (ΔS1Ea/S2Ea); PEPa (ΔPEPa); LVSTa(ΔLVSTa); HR (ΔHR); SBP (ΔSBP); MAP (ΔMAP); DBP (ΔDBP); SI (ΔSI); PPV (ΔPPV); and CVP (ΔCVP).
